# Phase I study of the indoleamine 2,3-dioxygenase 1 inhibitor navoximod (GDC-0919) as monotherapy and in combination with the PD-L1 inhibitor atezolizumab in Japanese patients with advanced solid tumours

**DOI:** 10.1007/s10637-019-00787-3

**Published:** 2019-05-24

**Authors:** Takahiro Ebata, Toshio Shimizu, Yutaka Fujiwara, Kenji Tamura, Shunsuke Kondo, Satoru Iwasa, Kan Yonemori, Akihiko Shimomura, Shigehisa Kitano, Takafumi Koyama, Natsuko Sato, Kiyohiko Nakai, Michiyasu Inatani, Noboru Yamamoto

**Affiliations:** 1 Department of Experimental Therapeutics, National Cancer Center Hospital, 5-1-1, Tsukiji, Chuo-ku, Tokyo, 1040045 Japan; 2 Department of Breast and Medical Oncology, National Cancer Center Hospital, Tokyo, Japan; 3 Chugai Pharmaceutical Co., Ltd., Tokyo, Japan

**Keywords:** Atezolizumab, Cancer, GDC-0919, Japanese, Navoximod, Phase I

## Abstract

Navoximod (GDC-0919) is a small molecule inhibitor of indoleamine-2,3-dioxygenase 1. This study investigated the safety, tolerability and pharmacokinetics of navoximod alone and in combination with atezolizumab in Japanese patients with advanced solid tumours. This was a phase I, open-label, dose-escalation study. Patients received monotherapy with navoximod 400 mg, 600 mg or 1000 mg orally twice daily (BID) in Stage 1 and navoximod 200 mg, 400 mg, 600 mg or 1000 mg orally BID plus atezolizumab 1200 mg intravenously every 21 days in Stage 2. Objectives included safety, tolerability, efficacy and pharmacokinetic outcomes.Overall, 20 patients were enrolled (Stage 1: *n* = 10; Stage 2: *n* = 10). No dose-limiting toxicities were observed. In Stage 1, treatment-related adverse events (TRAEs) of any grade that occurred in ≥20% of patients were chromaturia (50%) and maculopapular rash (20%). Grade ≥ 3 TRAEs were reported in two patients (20%; maculopapular rash and lipase increased). In Stage 2, TRAEs that occurred in ≥30% of patients were chromaturia (60%) and, decreased appetite (40%). Grade ≥ 3 TRAEs were reported in three patients (30%; hyponatraemia, aspartate aminotransferase increased, alanine aminotransferase increased, lymphopaenia and neutropaenia). Stable disease was observed in five patients (50%) in Stage 1 and eight patients (80%) in Stage 2. Navoximod showed linear pharmacokinetics. The recommended dose of navoximod monotherapy was determined as 1000 mg orally BID, and could be considered 1000 mg orally BID in combination with atezolizumab. Navoximod as monotherapy and in combination with atezolizumab was well tolerated in Japanese patients with advanced solid tumours.

**Electronic supplementary material:**

The online version of this article (10.1007/s10637-019-00787-3) contains supplementary material, which is available to authorized users.

## Introduction

Indoleamine-2,3-dioxygenase 1 (IDO1) is an intracellular enzyme that catalyses the first, rate-limiting step in the kynurenine pathway of tryptophan catabolism [1]. IDO1 creates a tryptophan-depleted microenvironment, which induces a starvation response in T cells and promotes regulatory T cell (Treg) differentiation. In addition, kynurenine and other downstream products of IDO1 promote Treg differentiation and induce an immunosuppressive phenotype in dendritic cells and macrophages [2]. As a result, IDO1 has local and systemic immunosuppressant and tolerogenic effects [1].

IDO1 is expressed in multiple tumour types, including melanoma, pancreatic adenocarcinoma, ovarian cancer, acute myeloid leukaemia, colorectal cancer, prostate cancer and endometrial cancer [3–9]. The level of IDO1 expression has been shown to predict poor outcomes in these cancers [3–9]. Due to its role in tumour immunosuppression, IDO1 represents an attractive target for cancer therapy [10].

Navoximod (GDC-0919) is an investigational small-molecule inhibitor of IDO1 characterised by oral bioavailability and a favourable pharmacokinetic profile, as evaluated in animal models and human participants [11]. Navoximod has been shown to inhibit IDO1 in vitro in cell-based assays. Following oral administration, navoximod reduced plasma and tissue kynurenine concentrations by approximately 50%, blocked IDO1-induced T cell suppression and restored T cell function [11].

The aim of this study was to evaluate the safety, tolerability and pharmacokinetics of navoximod alone and in combination with atezolizumab in Japanese patients with advanced solid tumours.

## Methods

### Design and patients

**Fig. 1:**
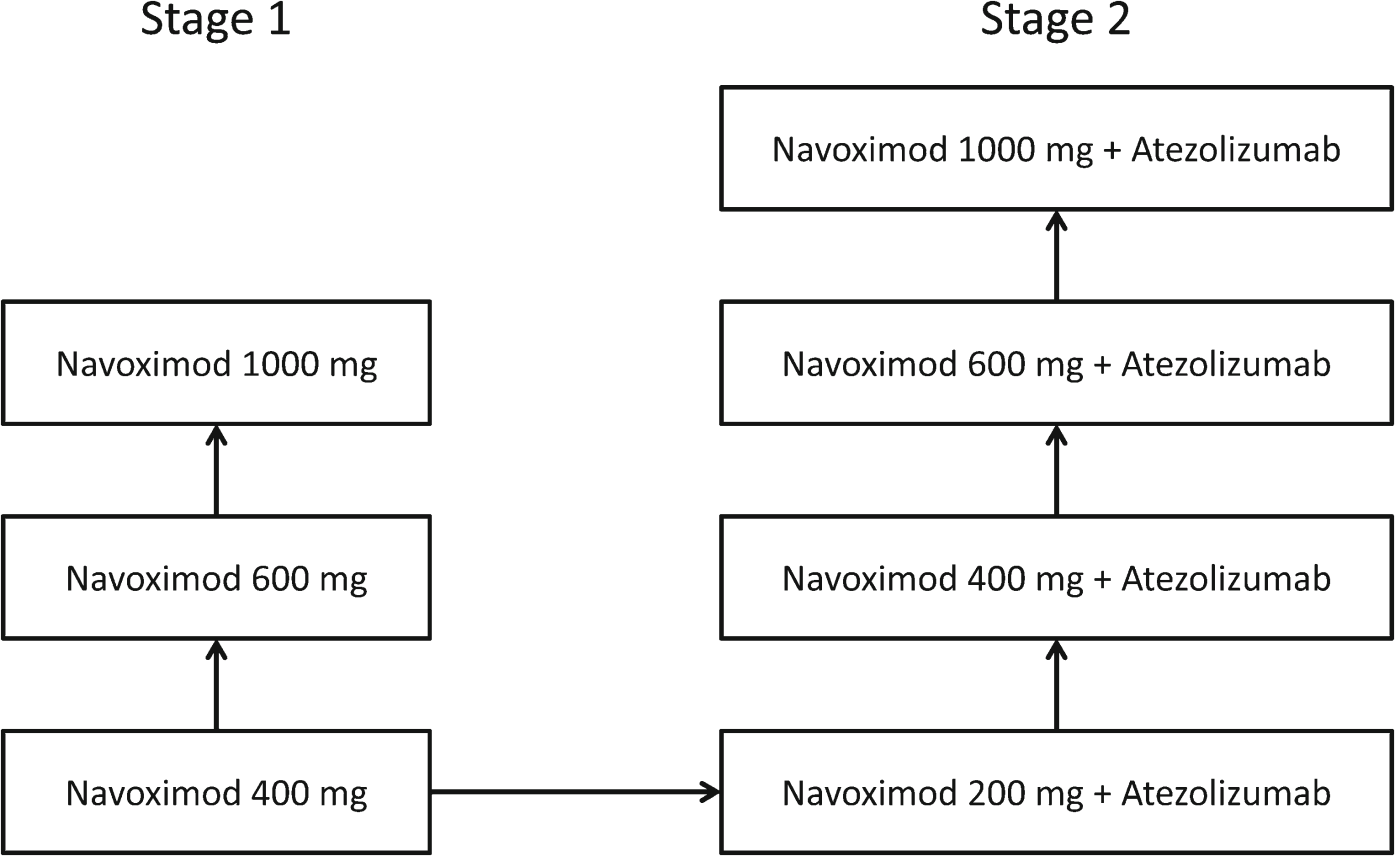
Study design

This was a phase I, open-label, single-centre, dose-escalation study conducted between August 2016 and February 2018 in Japanese patients with advanced solid tumours. The study consisted of two stages (Fig. 1). During Stage 1, to allow for pharmacokinetic evaluation, patients received a single initial dose of oral navoximod 400 mg, 600 mg or 1000 mg; 3–8 days after that, treatment with the same dose twice daily was initiated. Stage 2 was initiated after tolerability of the lowest dose of navoximod (400 mg) was confirmed in Stage 1. During Stage 2, patients received a single initial dose of oral navoximod 200 mg, 400 mg, 600 mg or 1000 mg, followed by twice-daily navoximod plus intravenous atezolizumab 1200 mg every 21 days.

The study protocol was approved by the institutional review boards of all participating centres and the study was conducted in accordance with the Declaration of Helsinki, Good Clinical Practice, and the Law for Ensuring the Quality, Efficacy, and Safety of Drugs and Medical Devices (paragraph 3 of article 14 and article 80–2). All study participants provided written informed consent before entering the study.

Patients were eligible for enrolment if they were aged ≥20 years, had a histologically or cytologically confirmed advanced or recurrent solid tumour refractory to standard therapy or for which no standard therapy existed, an Eastern Cooperative Oncology Group (ECOG) performance status of 0 or 1, life expectancy ≥12 weeks, radiographically evaluable lesions and adequate haematological and major organ function. Patients were required to complete surgery, radiotherapy, antibody therapy (except anti-programmed cell death protein [PD-1]/programmed death-ligand 1 [PD-L1] and anti-cytotoxic T lymphocyte–associated antigen [CTLA]-4 drugs), immunosuppressive therapy, live vaccines and other investigational drugs within 4 weeks of enrolment; chemotherapy within 3 weeks; anti-PD-1/PD-L1 antibody drugs within 10 weeks; anti-CTLA-4 antibody drugs and other cancer immunotherapy within 6 weeks; and blood transfusion, hematopoietic growth factors and endocrine therapy within 2 weeks. The main exclusion criteria were a history of hypersensitivity to an excipient of navoximod; gastrointestinal condition that could interfere with drug absorption; central nervous system or meningeal metastases that were symptomatic or required treatment; pleural effusion, pericardial effusion or ascites requiring drainage; ongoing grade ≥ 2 adverse reaction to a previous treatment (assessed according to the National Cancer Institute Common Terminology Criteria for Adverse Events [NCI CTCAE] version 4.03); previous grade ≥ 3 adverse reaction to an immunologically targeted anti-tumour drug; active infection requiring systemic treatment; current or previous autoimmune disease; current or previous clinically significant liver disease; and positive tests for human immunodeficiency virus, hepatitis B virus or hepatitis C virus. In addition, patients who had a history of hypersensitivity to drugs derived from Chinese hamster ovary cells or other recombinant human antibodies or recombinant proteins were excluded from Stage 2.

The target sample size was 6–18 patients (2–3 cohorts of 3–6 patients each) for Stage 1 and 9–24 patients (3–4 cohorts of 3–6 patients each) for Stage 2. Recruitment of patients to each dose level was sequential using the standard 3 + 3 study design. Patients could only be recruited to the next higher dose of navoximod if <1 patient developed dose-limiting toxicity (DLT) at the existing dose. At each dose level, treatment was continued until disease progression, DLT or other adverse event (AE) that would hinder the patient’s participation in the study, or until the patient requested discontinuation from the study.

Plasma samples were collected for analysis of navoximod and kynurenine/tryptophan levels according to the schedule outlined in Supplementary Table 1.

### Outcomes

Safety, pharmacokinetics, efficacy, exploratory pharmacodynamics and genetic outcomes were assessed in this study. The safety outcomes were the incidence, type, severity and time of onset of DLTs, AEs and treatment-related AEs (TRAEs). AEs were assessed using NCI CTCAE version 4.03. In addition, the maximum tolerated dose (MTD) was determined on the basis of DLTs. DLT was defined as one of the AEs listed in Supplementary Table 2 that occurred during the single dose administration period (2–7 days) and the first 21 days of twice daily navoximod administration in Stage 1 and Stage 2, and for which a potential causal relationship between navoximod and the AE could not be ruled out. MTD was defined as the highest dose at which <33% of patients experienced a DLT.

The pharmacokinetic outcomes were the plasma concentration of navoximod, area under the plasma concentration-time curve (AUC), peak plasma concentration (C_max_), time to C_max_ (T_max_) and plasma elimination half-life (t_1/2_). The efficacy outcomes were the response rate, disease control rate, progression-free survival (PFS) and duration of response. Efficacy outcomes were assessed using the Response Evaluation Criteria in Solid Tumours (RECIST) version 1.1. Plasma concentrations of kynurenine and tryptophan and their ratios and the presence of UGT1A1*6 and UGT1A1*28 polymorphisms were the exploratory outcomes of this study.

### Statistics

Safety endpoints were analysed in the safety population, which consisted of patients who received ≥1 dose of the study drug. Efficacy endpoints were analysed in the full analysis set, which consisted of patients who received ≥1 dose of the study drug and underwent ≥1 post-baseline efficacy assessment. A summary of AEs, TRAEs and DLTs by mapped term, appropriate thesaurus level and severity was provided for each cohort. Summary statistics, including mean, standard deviation, coefficient of variation, median, and minimum and maximum, were calculated for pharmacokinetic and pharmacodynamic parameters.

## Results

Between 31 August 2016 and 30 May 2017, 20 patients were enrolled and received navoximod (10 patients in Stage 1 and 10 patients in Stage 2; Table 1). Enrolment in Stage 2 was discontinued earlier than planned and only one patient was enrolled in the navoximod 1000 mg cohort. Patients were aged between 48 and 75 years. Diagnoses included thymic cancer, pancreatic cancer, small-cell lung cancer, adenoid cystic cancer of the palatal gingiva, leiomyosarcoma, cervical cancer, endometrial cancer, olfactory neuroblastoma, non-small–cell lung cancer, peritoneal cancer, ovarian cancer and bladder cancer. Most patients had ECOG performance status of 0 at baseline (*n* = 13, 65%).

**Table 1:** Baseline patient characteristics

	Stage 1				Stage 2				
	Navoximod 400 mg (*N* = 3)	Navoximod 600 mg (*N* = 4)	Navoximod 1000 mg (*N* = 3)	Stage 1 total (*N* = 10)	Navoximod 200 mg + Atezolizumab (*N* = 3)	Navoximod 400 mg + Atezolizumab (*N* = 3)	Navoximod 600 mg + Atezolizumab (*N* = 3)	Navoximod 1000 mg + Atezolizumab (*N* = 1)	Stage 2 total (*N* = 10)
Age, years									
Median	68	62	54	61.5	58	68	66	58	63.5
Range	64–74	48–75	52–59	48–75	57–70	56–70	61–69	–	56–70
Sex, n									
Male	2	2	0	4	1	2	3	1	7
Female	1	2	3	6	2	1	0	0	3
ECOG PS, n									
0	0	3	3	6	1	3	3	0	7
1	3	1	0	4	2	0	0	1	3
No. of prior systemic treatments, n									
Median	2	4	4	3	6	1	3	4	4
Range	2–3	2–6	1–11	1–11	4–15	1–6	3–4	–	1–15

*ECOG* Eastern Cooperative Oncology Group, *PS* performance status

### Safety

During Stage 1, TRAEs were reported in six out of 10 patients (60%; Table 2). Grade 3 TRAEs were reported in one patient (10%) who received navoximod 400 mg (maculopapular rash) and one patient (10%) who received navoximod 600 mg (lipase increased). The latter TRAE did not resolve after navoximod treatment was suspended, however, there were no other symptoms or abnormal findings. No grade 4 or 5 TRAEs were observed. In addition, no DLTs were observed during Stage 1 and the MTD was not reached. Based on these results, the recommended dose of navoximod monotherapy was determined as 1000 mg orally twice daily.

**Table 2:** Treatment-related adverse events reported in two or more patients during Stage 1

TRAE, n	Navoximod 400 mg (*N* = 3)	Navoximod 600 mg (*N* = 4)	Navoximod 1000 mg (*N* = 3)	Total (*N* = 10)
Any, n				
All grades	1	2	3	6
Grade ≥ 3	1	1	0	2
Chromaturia, n				
All grades	0	2	3	5
Grade ≥ 3	0	0	0	0
Maculopapular rash, n				
All grades	1	0	1	2
Grade ≥ 3	1	0	0	1

*TRAE* treatment-related adverse event

During Stage 2, TRAEs were reported in all 10 patients (100%; Table 3). Grade 3 TRAEs were reported in three patients (30%) and included hyponatraemia, lymphopenia, neutropenia and elevated AST and ALT. All grade 3 TRAEs were confirmed to have resolved. No grade 4 or 5 TRAEs were observed. During Stage 2, no DLTs were observed and the MTD was not reached. The recommended dose of navoximod in combination with atezolizumab was not determined because of early discontinuation; however, 1000 mg orally twice daily was well tolerated.

**Table 3:** Treatment-related adverse events reported in two or more patients during Stage 2

TRAE, n	Navoximod 200 mg + Atezolizumab (*N* = 3)	Navoximod 400 mg + Atezolizumab (*N* = 3)	Navoximod 600 mg + Atezolizumab (*N* = 3)	Navoximod 1000 mg + Atezolizumab (*N* = 1)	Total (*N* = 10)
Any					
All grades	3	3	3	1	10
Grade ≥ 3	1	2	0	0	3
Fatigue					
All grades	1	0	1	0	2
Grade ≥ 3	0	0	0	0	0
Chromaturia					
All grades	2	0	3	1	6
Grade ≥ 3	0	0	0	0	0
Decreased appetite					
All grades	1	1	1	0	3
Grade ≥ 3	0	0	0	0	0
Hyponatraemia					
All grades	1	1	0	0	2
Grade ≥ 3	1	1	0	0	2
AST increased					
All grades	0	2	0	0	2
Grade ≥ 3	0	1	0	0	1
ALT increased					
All grades	0	2	0	0	2
Grade ≥ 3	0	1	0	0	1

*ALT* alanine aminotransferase, *AST* aspartate aminotransferase, *TRAE* treatment-related adverse event

### Pharmacokinetics

**Fig. 2:**
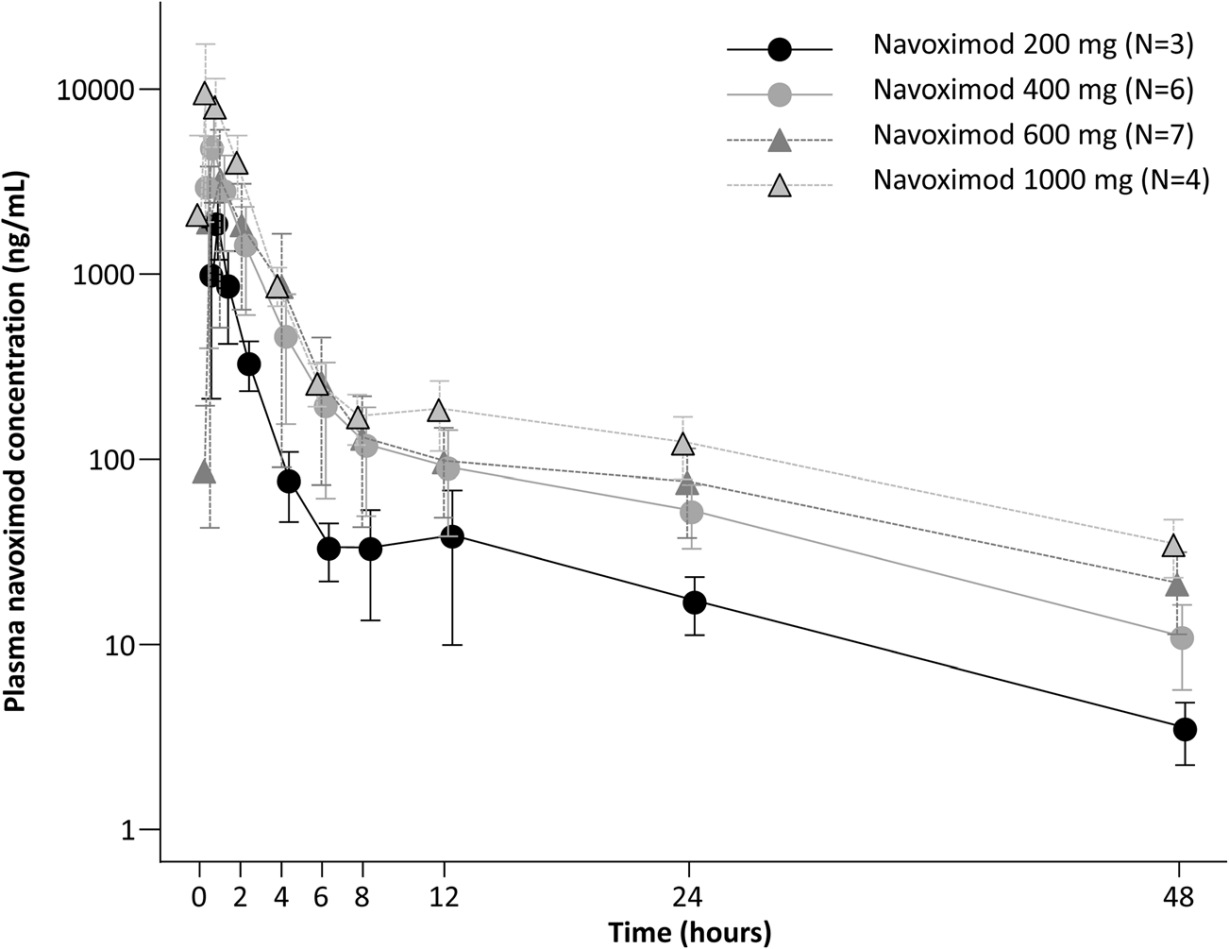
Plasma concentration of navoximod over time after single oral dose

After a single oral dose of navoximod, administered as monotherapy (Stage 1) or in combination with atezolizumab (Stage 2), the mean plasma concentration peaked at 15–60 min after administration and decreased precipitously after that (Fig. 2). When navoximod was administered alone in Stage 1, AUC and C_max_ changed dose-proportionally in the 400 mg, 600 mg and 1000 mg cohorts. Similar results were obtained when navoximod was administered in combination with atezolizumab in Stage 2.

**Fig. 3:**
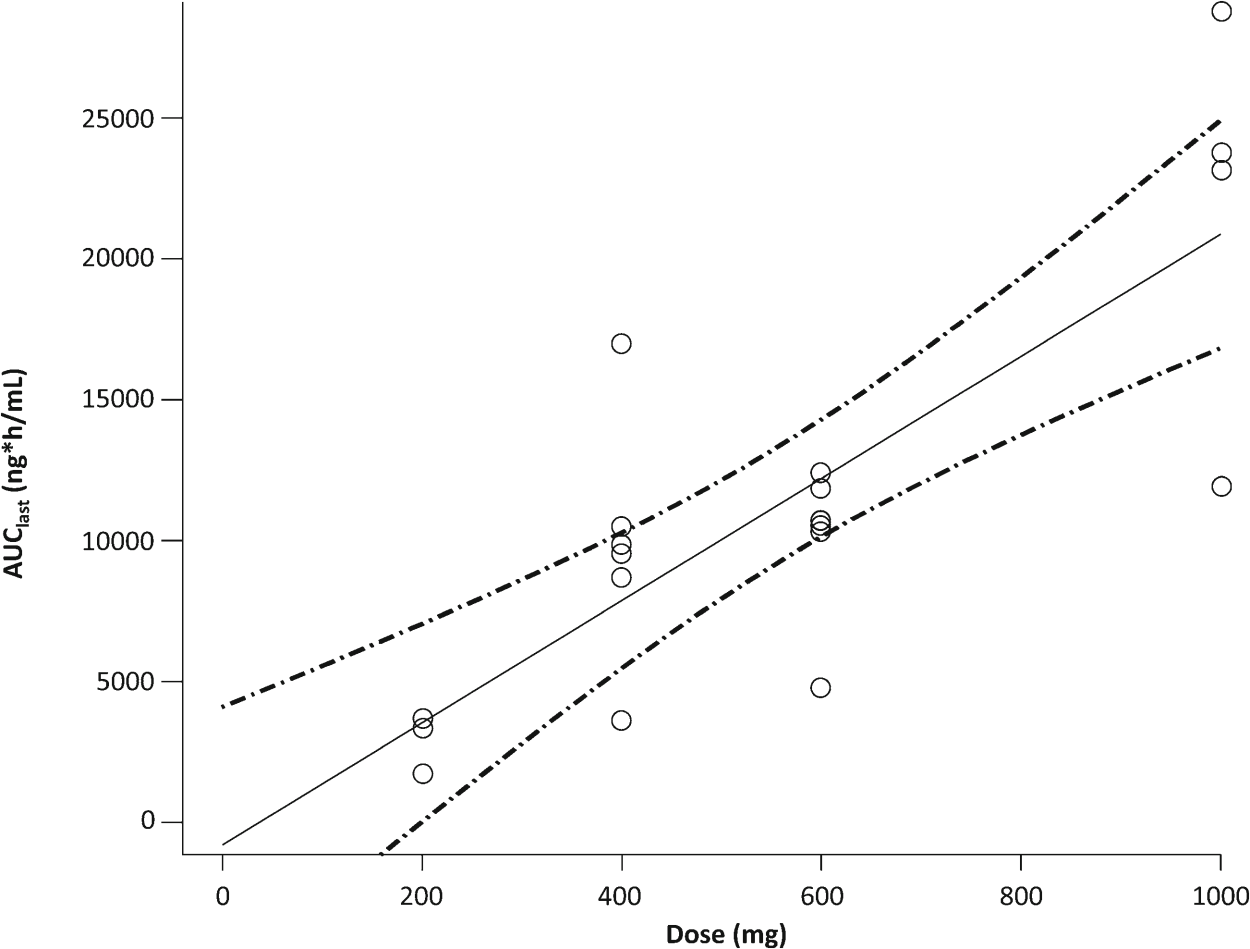
AUC after a single oral dose of navoximod AUC, area under the plasma concentration-time curve

Analysis of variance did not produce any statistically significant results. In linear regression analysis, the 95% confidence interval (95% CI) for the intercept of dose exposure contained 0 and the 95% CI for the intercept of the power model contained 1 (Fig. 3).

Dose-corrected navoximod exposure was similar in patients with UGT1A1 −/−, UGT1A1 −/*6, and UGT1A1 *6/*6; however, dose-corrected exposure was higher in patients with UGT1A1 −/*28.

**Fig. 4:**
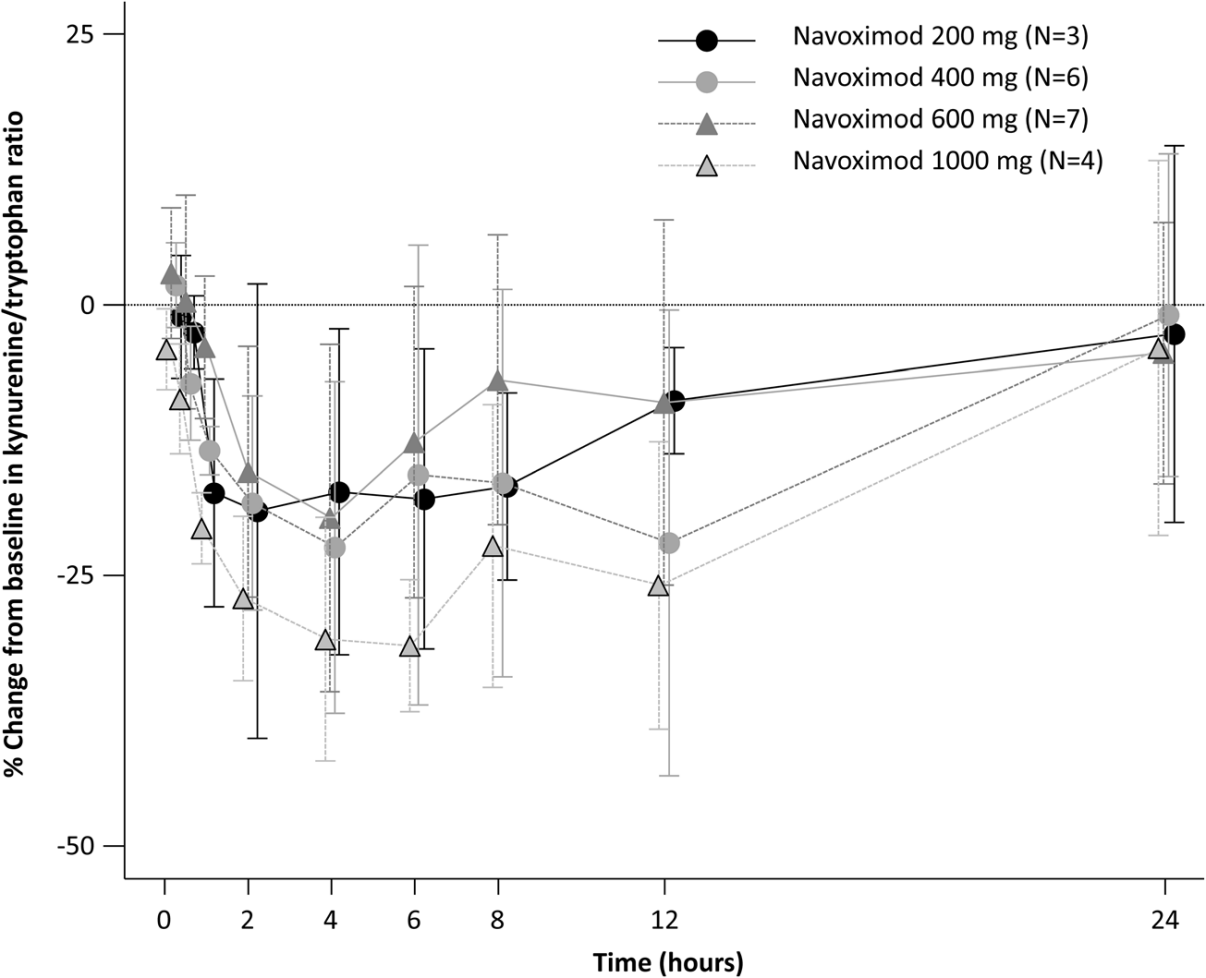
Percent change in plasma kynurenine-tryptophan ratio after single oral dose of navoximod

The change from baseline in kynurenine/tryptophan ratio was more marked with increasing doses of navoximod (Fig. 4).

### Efficacy

**Fig. 5:**
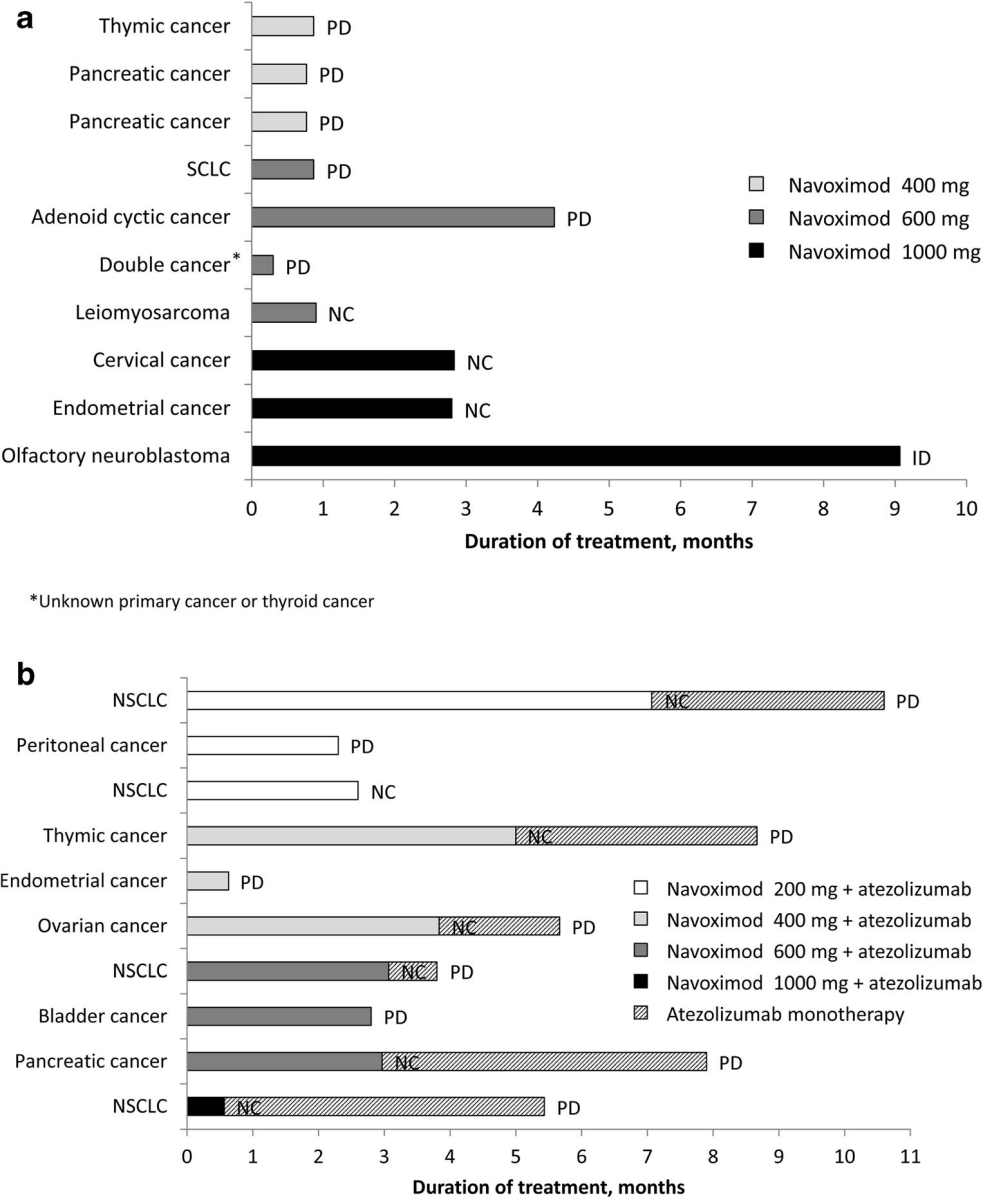
Time on treatment in **a** Stage 1; **b** Stage 2 ID, investigator’s decision; NSCLC, non-small-cell lung cancer; PD, progressive disease; SCLC, small-cell lung cancer; NC, non-compliant to the study treatment after being informed about discontinuation of navoximod development

Duration of treatment by cancer type in Stage 1 and Stage 2 are shown in Fig. 5a and b, respectively, along with the key reasons for navoximod discontinuation.

**Fig. 6:**
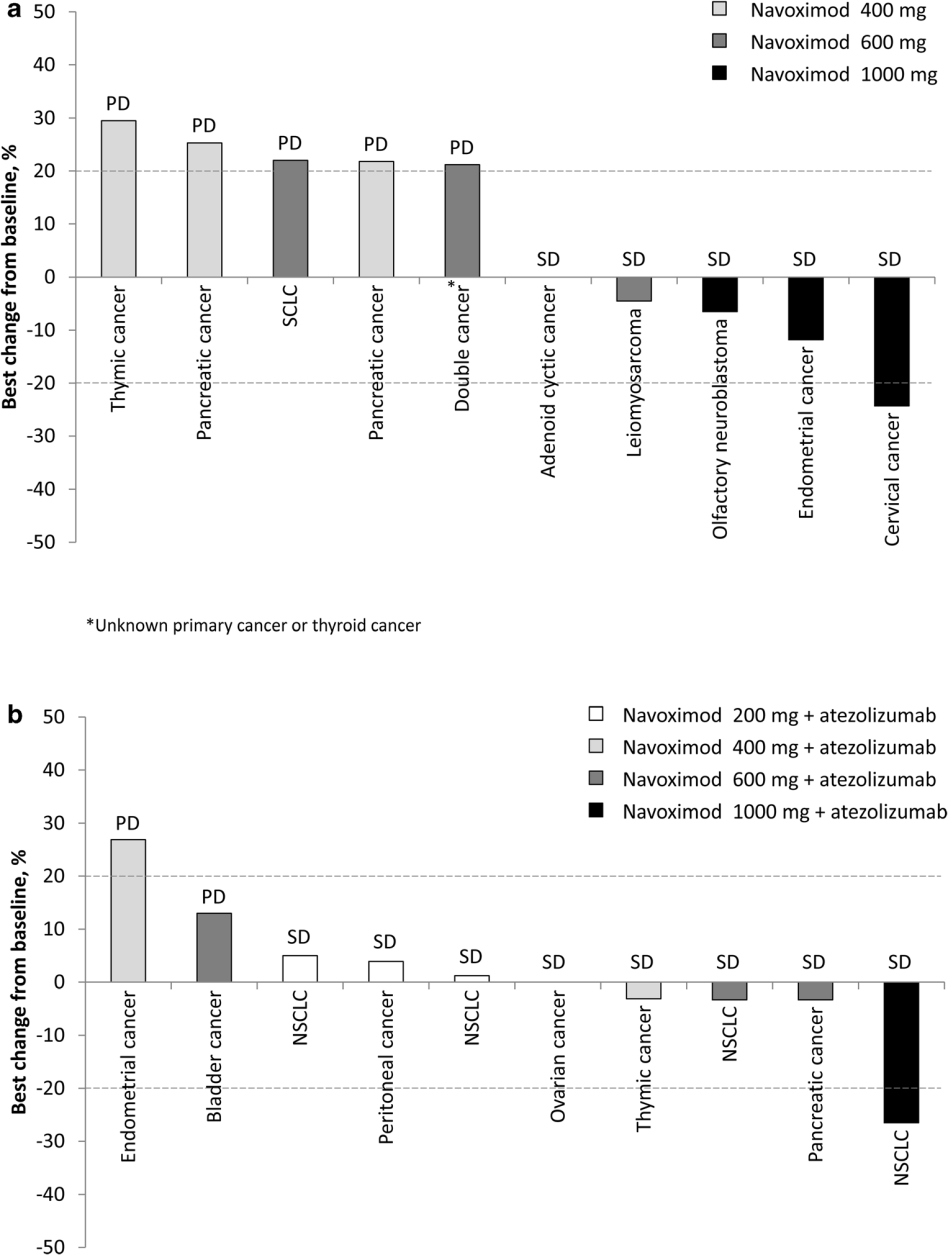
Best percent change from baseline in **a** Stage 1; **b** Stage 2 PD, progressive disease; SD, stable disease

During Stage 1, best overall response was stable disease (SD) in five patients (navoximod 600 mg: *n* = 2; navoximod 1000 mg: *n* = 3) and progressive disease (PD) in five patients (navoximod 400 mg: *n* = 3; navoximod 600 mg: *n* = 2; Fig. 6a). Complete response (CR) and partial response (PR) were not observed in any of the patients during Stage 1. Disease control was achieved in four out of 10 patients (40%). PFS ranged from 9 to 259 days.

During Stage 2, best overall response was SD in eight patients (navoximod 200 mg + atezolizumab: *n* = 3; navoximod 400 mg + atezolizumab: *n* = 2; navoximod 600 mg + atezolizumab: *n* = 2; navoximod 1000 mg + atezolizumab: *n* = 1) and PD in two patients (navoximod 400 mg + atezolizumab: *n* = 1; navoximod 600 mg + atezolizumab: *n* = 1; Fig. 6b). None of the patients had CR or PR during Stage 2. Disease control was achieved in eight out of 10 patients (80%). PFS ranged from 19 to 339 days.

## Discussion

The results of this phase I, open-label, single-centre, dose-escalation study indicate that navoximod, alone or in combination with atezolizumab, was well tolerated in Japanese patients with advanced solid tumours. No DLTs were observed during this study and MTD was not reached. As a result, the recommended dose of navoximod monotherapy was determined as 1000 mg twice daily. In combination with atezolizumab, the recommended dose of navoximod could not been determined because of early discontinuation of enrollment based on limited evidence of clinical activity in the clinical study conducted for the same time [12]. However, even though navoximod 1000 mg twice daily in combination with atezolizumab was administered in one patient, there was no safety concern, as such the navoximod 1000 mg twice daily in combination with atezolizumab 1200 mg every 21 days could be considered as recommended dose in our study.

The only symptomatic grade 3 or higher TRAE observed in this study was maculopapular rash (5%). The incidence of chromaturia was relatively high (55%); however, all events were asymptomatic. The mechanisms responsible for the development of chromaturia are not fully elucidated. Navoximod peak plasma concentration and exposure were dose-proportional in the 200–1000 mg range. In the present study, no PRs or CRs were observed with either navoximod monotherapy or navoximod plus atezolizumab combination. However, the majority of patients in both Stage 1 and Stage 2 had a best overall response of SD. Modest IDO1 inhibitory activity was observed with navoximod in the present study, as evidenced by reductions in the kynurenine/tryptophan ratio of up to approximately 50%. In the 200–1000 mg range, this activity increased in a dose-proportional manner.

The findings of this study are in line with those of the first-in-human study of navoximod monotherapy [13], and the combination study of navoximod and atezolizumab [12]. In the monotherapy study, 22 patients with advanced or recurrent solid tumours received monotherapy with navoximod 100 mg, 200 mg, 400 mg, 600 mg or 800 mg twice daily for 21 days, followed by 7 days without treatment, or 600 mg twice daily continuously. As in the present study, MTD was not reached. Grade 3 or higher AEs occurred in two patients (9%; lower gastrointestinal haemorrhage, diverticulitis), and dose interruptions and/or reductions were required in two patients. Similar to the present study, navoximod demonstrated linear pharmacokinetics with time to peak plasma concentration of approximately 60 min and a half-life of approximately 12 h. As in the present study, no CRs or PRs were observed. In patients who received navoximod 400 mg, 600 mg or 800 mg, plasma kynurenine levels decreased by approximately 25–30% at 2–4 h after administration [13]. In the combination study, 157 patients with advanced or recurrent solid tumours received navoximod at 6 dose levels (50–1000 mg twice daily continuously) in combination with atezolizumab. There was a single DLT of grade 3 sepsis syndrome at the 200 mg dose level. The tolerability of navoximod 1000 mg in combination with atezolizumab was confirmed and MTD was not reached. Grade 3 or higher treatment-related AEs occurred in 3 or more patients were rash (9%), fatigue (2%) and hepatitis (2%). The pharmacokinetics of navoximod in combination with atezolizumab was consistent with the pharmacokinetic observed in monotherapy. Sixteen patients (20%) achieved partial response or complete response. The administration of navoximod decreased plasma kynurenine: navoximod 1000 mg exceeded the IC50 of IDO1 in ~90% of the patients and decreases approximately 25% of plasma kynurenine. The efficacy of kynurenine modulation in this study was not so different from that of the combination study and as such, the recommended dose of navoximod in combination with atezolizumab based on the results of this study could be considered equal to that observed in the navoximod combination study.

Although navoximod was well tolerated in the present study and the monotherapy and combination therapy studies [12, 13], the efficacy of navoximod was modest. Previous studies with other IDO1 inhibitors have shown mixed efficacy results. In a phase I/II study in patients with advanced solid tumours, epacadostat plus nivolumab was associated with PR in some patients [14]. In a phase I/IIa study conducted in 29 patients with advanced bladder cancer, the combination of linrodostat plus nivolumab was associated with CR in one patient and PR in nine patients, resulting in the overall response rate of 34% [15]. Despite promising results of the phase I/II study, epacadostat in combination with pembrolizumab failed to show efficacy in a phase III, randomised, double-blind, placebo-controlled study conducted in 706 patients with unresectable or metastatic melanoma (ECHO-301/KEYNOTE-252) [16]. There were no significant differences between epacadostat plus pembrolizumab and placebo plus pembrolizumab in either PFS (hazard ratio [HR] = 1.00; 95% CI 0.83–1.21; *p* = 0.517) or overall survival (HR = 1.13; 95% CI 0.86–1.49; *p* = 0.807) [16]. Insufficient inhibition of kynurenine synthesis, inappropriate patient selection and crosstalk between PD-1/PD-L1 and IDO1 pathways could have contributed to these results.

Discovery of IDO1 stimulated research into the immunomodulatory effects of tryptophan, kynurenine and other metabolites of the kynurenine pathway, and this has improved our understanding of the immune system [1, 17]. However, preclinical studies have shown that IDO1 inhibitors are ineffective as stand-alone cancer treatments [13]. It should be remembered that inhibitors of PD-1 and PD-L1 have also shown modest activity when used as monotherapy. Due to the complexity of the immune system, it is likely that combinations of two or more immune checkpoint inhibitors will be necessary for effective cancer therapy [13]. Therefore, the therapeutic role of IDO1 inhibitors is likely to be as part of a combination regimen, rather than as monotherapy.

In conclusion, navoximod was well tolerated as monotherapy and in combination with atezolizumab in Japanese patients with advanced solid tumours. Additional research into the role of the IDO1 pathway in the maintenance of tumour microenvironment is necessary for further development of IDO1 inhibitors.

## Electronic supplementary material

ESM 1 (PDF 310 kb)
