# Supplementary material for: Phase I study of the indoleamine 2,3-dioxygenase 1 inhibitor navoximod (GDC-0919) as monotherapy and in combination with the PD-L1 inhibitor atezolizumab in Japanese patients with advanced solid tumours
Source: Invest New Drugs. 2019 May 24;38(2):468–77. doi: 10.1007/s10637-019-00787-3 (PMC7066107; doi:10.1007/s10637-019-00787-3)
Supplement: Supplementary file 1 — (PDF 310 kb) [file 10637_2019_787_MOESM1_ESM.pdf]

## Electronic supplementary material

**Supplementary Table 1.** Schedule for blood sampling for pharmacokinetic assessments

| Visit | Sampling time for navoximod pharmacokinetics (time window) | Sampling time for kynurenine/tryptophan pharmacodynamics (time window) |
|---|---|---|
| | **Stage I** | |
| Cycle 0 Day 1 | 5 min before dosing (−30 min)<br>15 min after dosing (±5 min)<br>30 min after dosing (±5 min)<br>1 hr after dosing (±5 min)<br>2 hr after dosing (±5 min)<br>4 hr after dosing (±10 min)<br>6 hr after dosing (±30 min)<br>8 hr after dosing (±30 min)<br>12 hr after dosing (±30 min) | 5 min before dosing (−30 min)<br>15 min after dosing (±5 min)<br>30 min after dosing (±5 min)<br>1 hr after dosing (±5 min)<br>2 hr after dosing (±5 min)<br>4 hr after dosing (±10 min)<br>6 hr after dosing (±30 min)<br>8 hr after dosing (±30 min)<br>12 hr after dosing (±30 min) |
| Cycle 0 Day 2 | 24 hr after dosing on Cycle 0 Day 1 (±2 hr) | 24 hr after dosing on Cycle 0 Day 1 (±2 hr) |
| Cycle 0 Day 3 | 48 hr after dosing on Cycle 0 Day 1 (±2 hr), before dosing on Cycle 1 Day 1 | – |
| Cycle 1 Day 8 | 5 min before dosing (−30 min) | – |
| Cycle 2 Day 1 | 5 min before dosing (−30 min)<br>15 min after dosing (±5 min)<br>30 min after dosing (±5 min)<br>1 hr after dosing (±5 min)<br>2 hr after dosing (±5 min)<br>4 hr after dosing (±10 min)<br>6 hr after dosing (±30 min)<br>8 hr after dosing (±30 min)<br>12 hr after first dose (±60 min), before second dose | 5 min before dosing (−30 min)<br>15 min after dosing (±5 min)<br>30 min after dosing (±5 min)<br>1 hr after dosing (±5 min)<br>2 hr after dosing (±5 min)<br>4 hr after dosing (±10 min)<br>6 hr after dosing (±30 min)<br>8 hr after dosing (±30 min)<br>12 hr after first dose (±60 min), before second dose |
| Cycle 4 Day 1 | 5 min before dosing (−30 min) | – |
| Cycle 9 Day 1 | 5 min before dosing (−30 min) | – |
| Last observation | Any time | – |
| | **Stage 2** | |
| Cycle 0 Day 1 | 5 min before dosing (−30 min)<br>15 min after dosing (±5 min)<br>30 min after dosing (±5 min)<br>1 hr after dosing (±5 min)<br>2 hr after dosing (±5 min)<br>4 hr after dosing (±10 min)<br>6 hr after dosing (±30 min)<br>8 hr after dosing (±30 min)<br>12 hr after dosing (±60 min) | 5 min before dosing (−30 min)<br>15 min after dosing (±5 min)<br>30 min after dosing (±5 min)<br>1 hr after dosing (±5 min)<br>2 hr after dosing (±5 min)<br>4 hr after dosing (±10 min)<br>6 hr after dosing (±30 min)<br>8 hr after dosing (±30 min)<br>12 hr after dosing (±60 min) |

| | | |
|---|---|---|
| Cycle 0 Day 2 | 24 hr after dosing on Cycle 0 Day 1 (±2 hr) | 24 hr after dosing on Cycle 0 Day 1 (±2 hr) |
| Cycle 0 Day 3 | 48 hr after dosing on Cycle 0 Day 1 (±2 hr), before dosing on Cycle 1, Day 1 | – |
| Cycle 1 Day 8 | 5 min before dosing (−30 min) | – |
| Cycle 2 Day 1 | 5 min before dosing (−30 min)<br>15 min after dosing (±5 min)<br>30 min after dosing (±5 min)<br>1 hr after dosing (±5 min)<br>2 hr after dosing (±5 min)<br>4 hr after dosing (±10 min)<br>6 hr after dosing (±30 min)<br>8 hr after dosing (±30 min) | 5 min before dosing (−30 min)<br>15 min after dosing (±5 min)<br>30 min after dosing (±5 min)<br>1 hr after dosing (±5 min)<br>2 hr after dosing (±5 min)<br>4 hr after dosing (±10 min)<br>6 hr after dosing (±30 min)<br>8 hr after dosing (±30 min) |
| Cycle 4 Day 1 | 5 min before dosing (−30 min) | – |
| Last observation | Any time | – |

Hr, hour; min, minutes

**Supplementary Table 2. Adverse events that could be considered dose-limiting toxicities**

| |
|---|
| 1. Grade 4 neutropenia persisting for ≥5 days or requiring treatment with G-CSF |
| 2. Febrile neutropenia |
| 3. Grade 4 thrombocytopenia or grade 3 thrombocytopenia requiring platelet transfusion |
| 4. Grade ≥4 anaemia |
| 5. Grade ≥3 non-hematologic toxicity (excluding transient electrolyte abnormalities) Abnormal liver function levels (total bilirubin, AST, ALT and ALP), diarrhoea, nausea, vomiting, and skin toxicity will be assessed as DLTs only if they meet the following criteria. Grade ≥3 laboratory abnormalities with exception of abnormal liver function levels will be assessed whether DLT or not, based on the comprehensive evaluation during Sponsor and Investigator about clinical finding and clinical course<br>a. Total bilirubin, AST, and ALT: A grade ≥3 increase persisting for ≥3 days will be assessed as a DLT. However, if the patient has a liver metastasis and the site reference range was exceeded at enrolment, an increase in total bilirubin of ≥5 times the site reference range or an increase in AST or ALT of ≥7.5 times the ULN will be assessed as a DLT<br>b. ALP: A grade ≥3 increase persisting for ≥3 days will be assessed as a DLT. However, if the patient has a bone or liver metastasis and the site reference range was exceeded at enrolment, an increase of ≥10 times the site reference range will be assessed as a DLT<br>c. Diarrhoea, nausea, vomiting, skin toxicity: A grade ≥3 event persisting for ≥1 week despite appropriate intervention |
| 6. Adverse events in Cycle 1 for which a causal relationship with navoximod or atezolizumab cannot be ruled out, and that require interruption of navoximod treatment for a total of ≥6 days |

ALP, alkaline phosphatase; ALT, alanine aminotransferase; AST, aspartate aminotransferase; DLT, dose-limiting toxicity; G-CSF, granulocyte-colony stimulating factor; ULN, upper limit of normal
